# Early detection of breast cancer: benefits and risks of supplemental breast ultrasound in asymptomatic women with mammographically dense breast tissue. A systematic review

**DOI:** 10.1186/1471-2407-9-335

**Published:** 2009-09-20

**Authors:** Monika Nothacker, Volker Duda, Markus Hahn, Mathias Warm, Friedrich Degenhardt, Helmut Madjar, Susanne Weinbrenner, Ute-Susann Albert

**Affiliations:** 1 Agency for Quality in Medicine, Berlin, Germany; 2 Department of Gynecology, Gynecological Endocrinology and Oncology, University of Marburg, Marburg, Germany; 3 Department of Gynecology and Obstetrics, University of Tuebingen, Tübingen, Germany; 4 Department of Gynecology and Obstetrics, University of Cologne, Cologne, Germany; 5 Women's hospital, Franziskus-Hospital, Bielefeld, Germany; 6 German Clinic for Diagnostics, Wiesbaden, Germany; 7 Agency for Quality in Medicine, Berlin, Germany; 8 Department of Gynecology, Gynecological Endocrinology and Oncology, University of Marburg, Marburg, Germany

## Abstract

**Background:**

Mammographic screening alone will miss a certain fraction of malignancies, as evidenced by retrospective reviews of mammograms following a subsequent screening. Mammographic breast density is a marker for increased breast cancer risk and is associated with a higher risk of interval breast cancer, i.e. cancer detected between screening tests. The purpose of this review is to estimate risks and benefits of supplemental breast ultrasound in women with negative mammographic screening with dense breast tissue.

**Methods:**

A systematic search and review of studies involving mammography and breast ultrasound for screening of breast cancer was conducted. The search was performed for the period 1/2000-8/2008 within the data source of PubMed, DARE, and Cochrane databases. Inclusion and exclusion criteria were determined prospectively, and the Oxford evidence classification system for diagnostic studies was used for evidence level. The parameters biopsy rate, positive predictive value (PPV) for biopsy, cancer yield for breast ultrasound alone, and carcinoma detection rate by breast density were extracted or constructed.

**Results:**

The systematic search identified no randomized controlled trials or systematic reviews, six cohort studies of intermediate level of evidence (3b) were found. Only two of the studies included adequate follow-up of subjects with negative or benign findings. Supplemental breast ultrasound after negative mammographic screening permitted diagnosis of primarily invasive carcinomas in 0.32% of women in breast density type categories 2-4 of the American College of Radiology (ACR); mean tumor size for those identified was 9.9 mm, 90% with negative lymph node status. Most detected cancers occurred in mammographically dense breast ACR types 3 and 4. Biopsy rates were in the range 2.3%-4.7%, with PPV of 8.4-13.7% for those biopsied due to positive ultrasound, or about one third of the PPV of biopsies due to mammography. Limitations: The study populations included wide age ranges, and the application to women age 50-69 years as proposed for mammographic screening could result in less striking benefit. Further validation studies should employ a uniform assessment system such as BI-RADS and report not only PPV, but also negative predictive value, sensitivity and specificity.

**Conclusion:**

Supplemental breast ultrasound in the population of women with mammographically dense breast tissue (ACR 3 and 4) permits detection of small, otherwise occult, breast cancers. Potential adverse impacts for women in this intermediate risk group are associated with an increased biopsy rate.

## Background

Up until the early 1990's, breast ultrasound examinations were primarily used to distinguish between cysts and solid breast masses and for image-guided, minimally invasive interventions [1-3], but the diagnostic potential of breast ultrasound has improved since then. Evolving sonographic technology with high-frequency transducers in the 7.5-10 MHz range and evolving knowledge has established breast ultrasound in the past few years as an imaging procedure to supplement mammography [4-6].

In order to ensure standardized evaluation criteria, the American College of Radiology (ACR) developed a Breast Imaging Reporting And Data System (BI-RADS) classification for breast ultrasound examinations in 2003 [7], which is analogous to the BI-RADS classification for mammography [8]. The classification for breast ultrasound (US BI-RADS) consists of seven categories: 1) negative, 2) benign, 3) probably benign, 4) suspicious abnormality, 5) highly suggestive of malignancy, and 6) known malignancy. Category 0 is assigned for results requiring additional imaging due to limited assessment. Several international comprehensive quality standards for equipment and staff performing breast ultrasound have adapted the US BI-RADS criteria [9-12].

International guidelines recommend that breast ultrasound be used as a supplemental examination but not as a primary method for screening of breast cancer [13-17].

Screening for breast cancer focuses on detecting occult cancer at an early stage with tumor size preferably smaller than 1 cm, negative lymph node status and with no evidence of distant spread [18]. Mammography has been established as the primary method for screening. Some 35%-45% of non-palpable cancers are detected as microcalcifications in mammographic studies [19]. These microcalcifications can sometimes be visualized by modern ultrasound equipment, but cannot be reliably identified as such without knowledge of mammography [20,21]. However, not every carcinoma is detected in breast cancer screening. Breast density is one of the factors leading to false-negative findings in mammography [22-25]. Furthermore, mammographically dense breast tissue has been identified as an independent marker strongly associated with breast cancer risk and in particular with higher risk of interval cancer, i.e. cancer detected between screening tests [26-28]. Epidemiological studies have confirmed that individuals at varying risks according to the appearance of dense breast tissue in mammograms can be identified, and there is strong evidence for influence by genetic variants [29,30].

This systematic review examines two issues: First, can a supplemental breast ultrasound examination performed as a second-line screening procedure after negative mammography improve the early detection rate of breast cancer in asymptomatic women with dense breast tissue? Second, what potential risks does this involve for the women examined?

## Methods

The aim was to identify studies that provide reliable information on the benefit (positive effects of intervention) and the potential risks (negative effects of intervention) of supplemental breast ultrasound in breast cancer screening. The criteria for inclusion or exclusion of studies were established prospectively before actually running a systematic search of the literature.

The study populations included asymptomatic primarily healthy women who took part in mammographic screening. The evaluation was not limited to women who had been invited within the framework of an organized mammography screening program.

The value of the intervention 'Doppler sonography of the breast or axilla' was not included in the questions studied. Histological confirmation or adequate follow-up were taken as standards of reference.

The Oxford Classification for diagnostic studies was used, and publication quality was reported separately when assessing the studies from a methodological perspective [31].

### Criteria for inclusion and exclusion

In order to ensure evaluability and comparability, the studies were required to contain the following information to be included in the present analysis:

- breast density according to the BI-RADS ACR categories and/or quantification; ACR 1: almost entirely fat (low density, up to 25% mammary gland parenchyma), ACR 2: scattered fibroglandular densities (average density, 26-50% gland parenchyma), ACR 3: heterogeneously dense (high density, 51%-75% gland parenchyma), ACR 4: extremely dense (very high density, more than 75% gland parenchyma) [8].

For inclusion, all of the following criteria were required to be satisfied:

1. study/review deals with the questions under investigation

2. adequate type of study

3. adequate study population

4. intervention complies with technical standards (transducer frequency > 5 MHz)

5. required data are provided

Publications were excluded if any of the following criteria were met:

1. redundancy: multiple publications of the same data

2. methods: case reports, expert opinions, or poor-quality case-control studies

### Literature search

The systematic literature search covered the period from January 1^st^, 2000 up to August 30^th^, 2008 in the following databases:

1. PubMed (Internet portal of the National Library of Medicine) http://www.ncbi.nlm.nih.gov/sites/entrez?db=pubmed

2. Database of Abstracts of Reviews of Effects (DARE), of the Centre for Reviews and Dissemination (CRD) http://www.york.ac.uk/inst/crd/crddatabases.htm#DARE

3. Cochrane-database 'Cochrane Reviews' and 'Clinical Trials' http://www3.interscience.wiley.com/cgi-bin/mrwhome/106568753/HOME

The following search words were used:

PubMed: screening AND (mammography OR mammogram) AND breast neoplasms

AND (ultrasonography, mammary OR breast echography)

Cochrane und DARE: mammography screening AND ultrasound

The abstracts found were checked for relevance of content. Abstracts that did not prove to be relevant were excluded. Full-text versions of the abstracts deemed to contain relevant information were checked as to the criteria for inclusion and exclusion. The reasons for excluding a full text were given in each case.

### Target values and their definition

Since none of the identified studies addressed the mortality rate or the difference in mortality rate contributed by supplemental breast ultrasound, surrogate parameters, namely the assessment criteria of diagnostic tests, the additional relative and absolute rates of detected cancers compared to mammography and tumor size and lymph node negative status were used as target values. Negative predictive value, sensitivity and specificity of breast ultrasound were only computed if the follow-up period continued as long as the screening interval, thus permitting the identification of false-negative findings.

The risk of adverse impacts for the women examined was quantified using the number of biopsies performed due to breast ultrasound findings. The positive predictive value for biopsies with malignant histological findings characterizes the number of unnecessary biopsies induced by false positives.

## Results

**Figure 1:**
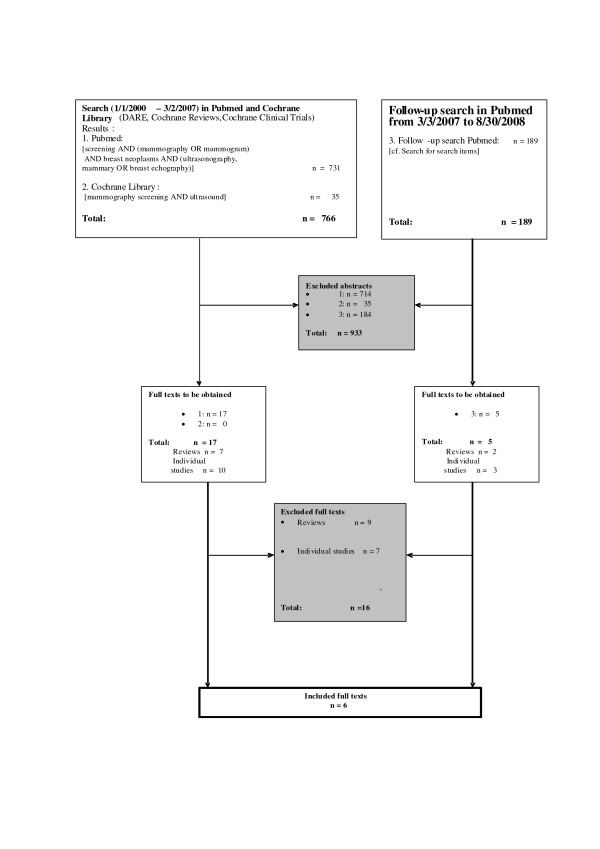
**Search strategy and search results**. Flow diagram of search strategy and search results.

The results of the literature search are represented in in Figure 1. and the details on the excluded studies are presented in Additional file 1[32-47]. Six cohort studies conforming to the inclusion criteria are discussed in detail (Additional file 2, Part 1; Additional file 3, Part II).

### Quality of studies and publications

It could be inferred from the information given in five of the six studies that women had been enrolled consecutively. The reference standard, i.e. histological confirmation of the findings, was applied in all studies for those results classified as malignant or suspicious or indeterminate.

Only Kolb et al., 2002 [48] reported a follow-up period of at least one year (up to the next screening exam) for all benign, but not for the negative findings. Kaplan, 2001 [49] mentioned a follow-up period for a part of the results classified as benign. None of the other studies provided any information on follow-up.

Due to non-consecutive inclusion of patients and/or an inconsistently applied reference standard, all studies were ultimately assigned to the level of evidence category 3b [31].

As for publication quality, it is worth noting that false positives were expressed in such a way that the positive predictive values for ultrasound examination and the biopsy rate could be calculated in only five of the six studies. Information on mean or median age was lacking in one study, while the relative rate of cancers detected by procedures done as a complement to mammography was reported in five of the six studies.

### Group of women examined

The systematic search yielded studies in which breast ultrasound was used as a supplemental examination following mammographic interpretation. Moreover, of the group of asymptomatic women with negative mammographic results, women with breast tissue density (ACR 2-4) went on to be examined by ultrasound. The only exception is the study performed by Leconte et al., 2003 [50], where 3% had palpable findings (Additional file 2, Part I).

The fraction of these women relative to all women screened within the specified period was reported in two studies at 36% (Kaplan, 2001 [49]) and 35.8% (Corsetti et al., 2008 [51]). The size of the study populations ranged from n = 1517 to n = 13547, with mean n = 5118.

### Age distribution

The median age was reported in four studies, ranging from 47.6 years to 60.7 years, with overall age ranges of more than 30 years in each study. One study provided information on mean age (52 years) [51] and one study just reported age ranges from 35-87 years (Kaplan., 2001 [49]).

### Cancer diagnosis according to breast density categories

Two studies analyzed mammographic results of breasts in categories ACR 3 to ACR 4 [51,49], and the other studies evaluated the mammograms of ACR 2 to ACR 4 breast tissue [48,50,52,53].

Women with breasts of types ACR 3 or ACR 4 proved to have the highest proportion of breast cancers diagnosed by ultrasound screening.

Leconte et al. [50] diagnosed 16 carcinomas in all; 11 of which were detected in ACR 3 and ACR 4 breast tissue and five in women with breasts in categories ACR 1 and 2. Buchberger et al. [53] diagnosed 36 malignancies in breast tissue of ACR types 3 and 4 (0.3% cancer detection rate ACR 3, 1.1% cancer detection rate in ACR 4), while two carcinomas were found in the ACR category 2 breasts (0.4%). Crystal et al. [52] found no carcinomas in ACR-2 women, and 0.4% and 0.3% in breast-density categories ACR 3 and ACR 4, respectively. Kaplan [49] diagnosed cancer at the rate of 0.11% in ACR 2 breast tissue and 0.27% and 0.25% in ACR categories 3 and 4, in the one series where technologists performed the screening ultrasound.

### Percentage of carcinomas identified in breast ultrasound

The relative percentage of carcinomas found in supplemental breast ultrasound examinations as a fraction of the total number of detected cancers was reported in four studies, with a mean percentage of 22.5% (15%-34%) [53,51,48,50]. The percentage as a fraction of the total population screened was calculated from all studies, with a mean value of 0.32% (0.23%-0.41%).

### Tumor size and stage (invasive/noninvasive and lymph node status)

The mean size of the detected carcinomas was 9.9 mm (median size range 9.0 mm to 11.0 mm) in five of the six studies [53,52,49,48,50]. The mean percentage of invasive cancer detected was 94% (81%-100%) compared to noninvasive cancer (ductal carcinoma in situ, DCIS) with a mean of 6% (0%-19%) in all studies. Lymph node status was reported in four studies with negative lymph nodes in 90% (86%-100%) [51,52,49,48].

### Quality of testing

The positive predictive value with regard to the detection of additional malignancies for ultrasound prompted biopsies was reported or was able to be inferred from the given data in four of the six studies. The mean positive predictive value was 15% (2%-28%) [53,52,49,48]. In other words, the percentage of positively classified findings for which no carcinoma was subsequently found ranged from 72% to 98%. This large variation of positive predictive values can mainly be attributed to the application of different sonographic criteria for malignancy and different assessment categories (see next section). Only one study reported that all findings classified as benign were followed up in the interval between two screenings (Kolb et al., 2002 [48]). However, Kolb et al. [48] do not mention a follow-up for those patients with negative results. Kolb states a sensitivity of 75.3%, a specifity of 96.8% and a negative predictive value of 99.7% [48].

### Assessment categories for breast ultrasound

The positive predictive value of detecting carcinomas by breast ultrasound mainly derives from the sonographic assessment criteria and categories applied. Overall, the range is still low relative to mammography.

Kaplan, 2001 [49] used a two-armed categorization approach, namely a simple subdivision into negative and positive. All positive results were considered to be potentially suspicious. In contrast to the other studies, Kaplan [49] also deemed cysts positive if they exceeded a size of 1 cm. The author had a low positive predictive value of 2% [49]. Three studies (Buchberger et al., 2000 [53]; Kolb et al., 2002 [48] and Leconte et al., 2003 [50]) subdivided their findings into three categories, but applied different definitions of these categories. Categories 1, 2 and 3 were called 'normal', 'benign', and 'suspicious' (Kolb et al. [48]) or 'benign', 'indeterminate' and 'malignant' (Buchberger et al. [53]). These authors found positive predictive values of biopsy of 10.3% (Kolb et al. [48]) and 28% (Buchberger et al. [53]). The values for Leconte's study could not be ascertained [50]. Crystal et al., 2003 [52] used four arms of classification, thereby reaching a positive predictive value for malignancy of 20%. Other than Buchberger et al., Crystal et al. included the 'indeterminate' findings in their calculation of suspicious findings. Only one study used the five categories proscribed by the BI-RADS classification for breast ultrasound scans (Corsetti et al., 2008 [51]). In this study, however, the positive predictive value could not be determined.

### Rate of biopsies as a result of breast ultrasound

The rate of biopsies resulting from suspicious findings detected by breast ultrasound ranged from 2.3% (Crystal et al. [52]) to 4.7% (Corsetti et al., 2008 [51]) as a fraction of the total population of women screened. The positive predictive value of biopsies ranged from 8.4% to 13.7%. The difference compared to the positive predictive value of the breast ultrasound categorization may be explained by the fact that biopsies were sometimes also performed when findings were classified as 'indeterminate'. Clinically speaking, this means that 86.3% up to 91.6% of the invasive interventions done to confirm the diagnosis did not lead to a diagnosis of cancer.

## Discussion

### Methodological aspects

Despite choosing a sensitive search strategy and a very broad definition of "breast cancer screening", only few single-center cohort studies analyzing breast ultrasound in the framework of breast cancer screening could be identified. All of these studies, however, were planned and conducted prospectively. The study populations were characterized by wide age ranges. For this reason, the results cannot be directly applied to women asked to participate in an organized population-based mammography screening program while aged between 50 and 69 years.

Since the studies applied different assessment criteria and classifications with regard to ultrasound morphology, their results are comparable only to a limited extent. As most studies lacked the requisite follow-up needed for computing sensitivity, specificity and negative predictive value, a comprehensive appraisal of the test quality of the intervention breast ultrasound could not be undertaken. None of the studies of US in breast screening were RCTs; therefore studies were not designed to provide evidence on screening benefit in terms of mortality reduction.

### Discussion of content

The effectiveness of breast ultrasound as a screening tool was mainly studied in women with breast tissue of the categories ACR 2 to ACR 4 and invariably only after previous mammography screening had yielded a negative result. Women with dense breasts run a four- to six-fold higher risk of developing breast cancer than other women [26,27]. The results revealed that supplemental breast ultrasound after negative mammographic screening permits the diagnosis of primarily invasive carcinomas in this risk group of women in an absolute mean of 0.32% of the cases. For comparative purposes: In population-based screening using mammography alone a mean of 0.4%-0.9% of all women screened were diagnosed with cancer [54].

The majority of cancers were detected in breast tissue of ACR types 3 and 4. According to these studies, the mean size of the cancers diagnosed additionally by breast ultrasound was not larger than the size of those visualized in mammography screening.

Berg (2004 [39]) reported similar results in a review of the application of breast ultrasound in women with dense breasts. The review was excluded because no systematic search strategy was specified, but Berg analyzed five of the six studies identified (Buchberger et al. [53]; Kaplan [49]; Kolb et al. [48]; Crystal et al. [52]; and Leconte et al. [50]) and, in addition, a study by Gordon et al., 1995 [55]. She calculated a pooled value for the additional detection of cancers in the study populations of 0.35% and reported that 94% of the cancers detected in breast ultrasound were invasive with a mean diameter of 9-11 mm [39]. More than 90% of the women in whom cancer was detected by breast ultrasound had breast density corresponding to ACR categories 3 or 4 [39].

Hence, the contribution of supplemental breast ultrasound for the detection of breast cancer is primarily relevant for women with ACR 3 or ACR 4 tissue density. The question of whether improved detection of carcinomas leads to decreased breast cancer mortality cannot be answered on the basis of the available studies, although mathematical models allow to relate tumor size, lymph node involvement and death rates [18,56,57].

Concerning possible adverse impacts of additional breast ultrasound, the biopsy rates in the evaluated studies of 2.3%-4.7% were significantly higher than the biopsy rates of about 1%-2% resulting from mammographic screenings [58-60]. Moreover, the positive predictive value of biopsies (mean 10.3%) was significantly lower than that of mammography screening (mean approximately 38%) [59-61], i.e. three times more women need to undergo a biopsy per carcinoma detected. Although findings visualized by ultrasound examination can easily be biopsied in a minimally-invasive procedure, risk assessment should still take into consideration the psychological strain that women experience before and during biopsy performed because of a false-positive ultrasound result [62,63].

The heterogeneous nature of the assessment criteria for breast ultrasound examinations, which in turn influence biopsy rates, is quite striking. Due to this heterogeneity, comparability of the studies is limited, and the effect of differences in experience of examiners is difficult to quantify. Since few studies included adequate follow-up of subjects with negative or benign findings, the false-negative rate in women with high breast density -- and hence the proportion of cancers missed by mammography and also missed by the addition of breast ultrasound in this group -- cannot be reliably estimated.

In 2003, Berg [61] initiated a prospective multicenter trial, randomized to the sequence of performance of mammography and ultrasound in women with intermediate and high breast cancer risk. Adding a single screening ultrasound to mammography yielded additional 1.1 to 7.2 cancers per 1000 high-risk women, but also substantially increased the number of false positives [37]. The study provided evidence for the importance of supplemental breast ultrasound screening. Furthrmore the standardized scanning and interpretive criteria proved to be practicable for independent performance and intrepretation and could be used for further implementation. However, in view of the selected study cohort the results cannot be applied directly to a population-based screening program for asymptomatic women aged 50-69 years without personal history evaluation prior performing mammography.

## Conclusion

As far as the transferability of the findings to a population-based mamographic screening program for women aged 50-69 years as currently practiced in many countries is concerned, one can conclude the following:

1. The reviewed studies provided limited evidence that within the framework of screening for breast cancer, an additional ultrasound examination after a negative mammogram is useful for the detection of primarily invasive cancers in women with mammographically dense breast tissue (ACR types 3 and 4, with more than 50% parenchymal gland), with the mean size of invasive cancers thus identified being 9.9 mm and in 90% with negative lymph node status.

2. As for adverse impacts resulting from the breast ultrasound intervention, three times more women need to undergo a biopsy per carcinoma detected by supplemental ultrasound compared to cancers detected by mammography screening alone.

3. Since the study populations were characterized by very broad age ranges and invariably also included younger women, the impact in an organized population-based mammography screening program, including only women in the age group of 50 to 69 years, could be reduced.

4. There is a need for prospective validating studies of risk-adjusted, second-line, supplemental breast ultrasound screening in women with dense breast tissue (ACR types 3 and 4), performed within the framework of established population-based mammography-screening-programs.

5. Validating studies should not only state the positive predictive value, but also the sensitivity, specificity and negative predictive value for breast ultrasound. Quality of performance standards and a uniform assessment system, such as the ultrasound BI-RADS™ categories, should be applied, so that the precise reasons for the biopsies can be given and explained accurately.

6. In analogy to the quality assurance assessments of established mammography screening programs, performance indicators and surrogate end points, such as tumor size, lymph node status are effective and accurate, and should be used for health care outcomes analyses. Beside lethality, issues such as quality of life and health care costs are of importance.

## Abbreviations

ACR: American College of Radiology; ADH: Atypical ductal hyperplasia; AUC: Area under ROC curve; BI-RADS: Breast Imaging Reporting and DataSystem™; CI: confidence interval; DARE: Database of Abstracts of Reviews of Effects; DEGUM: German Association for Ultrasound in Medicine; LCIS: lobular carcinoma in situ; LN: lobular neoplasia; MHz: Mega-Hertz; PPV: positive predictive value; ROC: receiver operating characteristic (sensitivity vs. specificity).

## Competing interests

The authors declare that they have no competing interests.

## Authors' contributions

MN was responsible for the methodology including search strategy. USA and MN reviewed abstracts and original work. MN and SW prepared the manuscript. MN and USA were responsible for data analyses. USA was responsible for manuscript editing. VD, MW, MH, HM, are expert members of the guideline task force group for breast ultrasound who reviewed the original work and made substantial contributions to interpretation. All authors read and approved the final manuscript.

## Pre-publication history

The pre-publication history for this paper can be accessed here:

http://www.biomedcentral.com/1471-2407/9/335/prepub

## Supplementary Material

Additional file 1 **Excluded reviews and individual studies**. Author, year, country; contents and reasons for exclusion. Click here for file

Additional file 2 **Evidence table of individual studies on supplemental ultrasound in breast cancer screening (Part I)**. Overview of individual studies with description of population, remarks to the results, conclusion and level of evidence according to the Oxford classification for diagnostic studies. Click here for file

Additional file 3 **Evidence table of individual studies on supplemental ultrasound in breast cancer screening (Part II)**. Detailed analysis of parameters for diagnostic accuracy. Click here for file
