# Supplementary material for: Early detection of breast cancer: benefits and risks of supplemental breast ultrasound in asymptomatic women with mammographically dense breast tissue. A systematic review
Source: BMC Cancer. 2009 Sep 20;9:335. doi: 10.1186/1471-2407-9-335 (PMC2760575; doi:10.1186/1471-2407-9-335)
Supplement: Additional file 1 — Excluded reviews and individual studies. Author, year, country; contents and reasons for exclusion. [file 1471-2407-9-335-S1.DOC]

| Table 1: Excluded reviews and individual studies | | |
| --- | --- | --- |
| Author, year, country | Contents | Reasons for exclusion |
| **1. Reviews** | | |
| Zonderland,  2000, Netherlands | Review of the entire range of indications for breast ultrasound | Narrative; one section about screening |
| Delorme,  2001,  Germany | Review of breast ultrasound and magnetic resonance imaging as supplementary procedures in mammography screening | Narrative; with regard to breast ultrasound, the focus is on studies that investigated criteria for malignancy |
| Gordon,  2002, USA | Review on breast ultrasound as an imaging procedure in screening for breast cancer | Narrative, but all relevant studies up to the year 2002 are discussed |
| Mehta,  2003, USA | Review of the entire range of indications for breast ultrasound | Narrative |
| Berg,  2004, USA | Review of supplemental ultrasound in breast cancer screening of women with dense breast tissue | No search strategy specified, but all relevant studies up to the year 2004 are discussed |
| Smith et al.,  2004, USA | Review on imaging procedures used in screening for breast cancer | Narrative; breast ultrasound discussed very briefly only; focus is on magnetic resonance tomography |
| Elmore et al.,  2005,  USA | Review of different mammography screening techniques, inter alia digital mammography and use of breast ultrasound. Systematic literature search | Studies of women with elevated risk of breast cancer and women with high mammographic breast density discussed together; no precise report on the respective results |
| Villeirs,  2007, Belgium | Review on supplemental breast ultrasound in breast cancer screening for women with dense breasts | Narrative |
| Nemec et al,  2007, USA | Review on techiques ( mammography, magnetic resonance tomography, breast ultrasound) for breast cancer screening | Narrative |
| **2.Individual Studies** | | |
| O’Driscoll et al.,  2001, UK | Prospective cohort study on the value of breast ultrasound in women with a moderately elevated familial risk of breast cancer (n= 149) | Special population; small no. of cases |
| Hou et al, 2002, Taiwan | Retrospective cohort study comparing clinical breast examination, mammography and ultrasound (n= 935). Result: Ultrasound sensitivity 90.4%, specificity 86.3%, abnormal screens 12.9%, biopsy rate 2.5%, cancer detection rate 2.0%, mean tumor size 1.2 cm. | Special and selected cohort; Asian women with female relatives with breast cancer; index cases from one hospital, no analysis of ACR. |
| Geller et al., 2005,  USA | Retrospective cohort study  Analysis of supplementary imaging procedures as part of the screening – no distinction between supplemental mammography or ultrasound examinations. Result: supplemental imaging lowers false-positive results and increases the number of false-negative findings. | No separate evaluation of breast ultrasound possible. |
| Ohlinger et al.,  2006,  Germany | Prospective cohort study on the benefit of using breast ultrasound as the first diagnostic procedure in asymptomatic patients (n= 448)  Result: Three T1 tumors were detected by ultrasound, however, these were also found in the subsequent mammographic examination. | No analysis of the added benefit of a breast ultrasound exam following mammographic screening. |
| Corsetti et al, 2006 | Prospective cohort study on the value of additional breast ultrasound versus mammography alone in women with dense breasts (n= 6449)  Result: Cancer detection rate was 0.44% or 17.3% of total cancer. After retrospective review, occult status was confirmed in 15 cases. | Double publication, study population is part of the included study Corsetti et al 2008. |
| Brancato et al., 2007, Italy | Cohort study on the benefit of ultrasound after negative mammography with high breast density. 52.3% of the total series with mammographic density of ACR 3-4. Subset of n = 5227 (20.3%) had ultrasound within 4 weeks after mammography screening (n= 25665) with negative findings and breast density in categories BI-RADS ACR 3-4 out of a series of n = 49044.  Result: The cancer detection rate was 0.002% for women 40-49, 0.07% for women 50-69. 7 women were retrospectively excluded with clinical findings. | High selection bias (only 20.3% of the population included) by including only women with self referral within four weeks – special study population |
| Berg et al, 2008, USA | Randomized study of the sequence of imaging: combined breast cancer screening with mammography and breast ultrasound in women with Intermediate to high risk for breast cancer (n=2725), with one year follow-up of all participants.  Result: The combination of the two imaging procedures compared to a mammogram alone resulted in an absolute sensitivity increase of 27.5% (p=0.003) and a specificity decrease of 6.1% (p<0.001). Diagnostic accuracy increased from AUC of 0.78 for mammography alone to 0.91 for the combination of the procedures. | Special and selected cohort: 53% women with a personal history of breast cancer, 19% women with lifetime risk for breast cancer >25% by Gail or Claus models, 12.5% 5-year risk for breast cancer according to Gail ≥ 2.5% and 0.9% BRCA1 or BRCA2 mutations carriers and women with precursor lesions.. . |
