# Supplementary material for: Early detection of breast cancer: benefits and risks of supplemental breast ultrasound in asymptomatic women with mammographically dense breast tissue. A systematic review
Source: BMC Cancer. 2009 Sep 20;9:335. doi: 10.1186/1471-2407-9-335 (PMC2760575; doi:10.1186/1471-2407-9-335)
Supplement: Additional file 2 — Evidence table of individual studies on supplemental ultrasound in breast cancer screening (Part I). Overview of individual studies with description of population, remarks to the results, conclusion and level of evidence according to the Oxford classification for diagnostic studies. [file 1471-2407-9-335-S2.DOC]

| **Table 2: Evidence table of individual studies on supplemental ultrasound in breast cancer screening** (Part I) | | | | | | |
| --- | --- | --- | --- | --- | --- | --- |
| **Author/ year/**  **country** | **Description of the cohort study** | | **Population** | **Remarks to results** | **Conclusion** | **LoE*** |
| Buchberger  et al.,  2000,  Austria | Data collection: | Prospective | n= 8103 with breast tissue density of types BI-RADS ACR 2-4, negative mammography and palpation underwent ultrasound, all positive ultrasound findings were analyzed, n= 405. | Time needed for ultrasound: 10-15 minutes  54% (n= 37) of the patients in whom breast ultrasound revealed cancer had a personal history of breast cancer. | Supplemental breast ultrasound screening recommended for women with dense breast tissue (ACR categories 2-4). | 3b |
|  | Period studied: | 1/1996-1/2000 |  |  |  |  |
|  | US-Performer: | N=1, specialized physician |  |  |  |  |
|  | US frequency: | 5-10 or 5-12 MHz |  |  |  |  |
|  | Scoring | 1. benign 2. indeterminate  3. malignant |  |  |  |  |
|  | US criteria: | Specified |  |  |  |  |
|  | Follow-up: | No data |  |  |  |  |
| Corsetti et al, 2008,  Italy | Data collection: | Prospective | N= 9157 (35.8%) of a total n = 25572 in the period studied with breasts density categories BI-RADS ACR 3 and 4 and normal mammography. | N=13 women with clinical symptoms were excluded retrospectively.  Therefore data was extracted for review analyses for asymptomatic women n = 9144.  Recall because of breast ultrasound: 4.8% (436/9144). False-positives because of breast ultrasound 0.9% (83/9144). In retrospect, 24.2% (8/33) were classified as false-negative mammograms. FNA was used instead of biopsy in 64% (279/436). | US detects early stage cancers in women with mammography-negative dense breast (ACR 3 -4).  US caused additional intervention and costs. | 3b |
|  | Period studied: | 1/2000-2/2007 |  |  |  |  |
|  | US-Performer: | N = 6; specialized physician (2 radiologists, 4 senologists) |  |  |  |  |
|  | US frequency: | 5-10 MHz |  |  |  |  |
|  | Scoring | US BIRADS |  |  |  |  |
|  | US criteria: | US BIRADS |  |  |  |  |
|  | Follow-up: | No data |  |  |  |  |
| Crystal et al.,  2003,  Israel | Data collection: | Prospective | n= 1517 within the period studied with breast tissue density of types BI-RADS ACR 2-4 and normal mammography screening and palpation. | Retrospective Analysis: 57% (n=4) of the women (n= 7) with cancers detected in breast ultrasound either had a personal history of breast cancer or were at familial high risk. Therefore data was extracted without high risk women n = 1199. | Breast ultrasound is beneficial in patients with dense breasts  (ACR categories 2-4) | 3b |
|  | Period studied: | 1/2000-1/2002 |  |  |  |  |
|  | US-Performer: | n= no data‚ specialized physician |  |  |  |  |
|  | US frequency: | 5-12 MHz |  |  |  |  |
|  | Scoring | 1.normal, 2.probably benign, 3.indeterminate, 4.malignant |  |  |  |  |
|  | US criteria | Specified |  |  |  |  |
|  | Follow-up: | No data |  |  |  |  |
| Kaplan, 2001,  USA | Data collection: | Prospective | n= 1862 within the period studied with breast density of BI-RADS categories ACR 3 and 4 | Median time needed for breast ultrasound 10 minutes (7-20).  Values do not agree with the study, since calculations were done per cancer and not per patient in the study itself. One of the n= 5 patients in whom carcinoma was detected by breast ultrasound had a familial high risk.  12-month follow-up for 72 patients revealed no additional carcinomas. | Breast ultrasound is helpful in women with dense breasts.  (ACR categories 3-4) | 3b |
|  | Period studied: | 7/1998-4/2000 |  |  |  |  |
|  | US-Performer: | n= no data ‚ specialized technologists |  |  |  |  |
|  | US frequency: | 7-12 MHz |  |  |  |  |
|  | Scoring | 1. negative, 2. positive |  |  |  |  |
|  | US criteria: | specificied for positive |  |  |  |  |
|  | Follow-up: | For benign 12 months |  |  |  |  |
| Kolb et al.,  2002,  USA | Data collection: | Prospective | n= 4897 of n= 5418 women in the period studied with breast tissue density of BI-RADS ACR 2-4 with normal mammograms and physical examination. | Median time needed for breast ultrasound: 4.5 minutes (2-11.5).  12-months follow-up for benign findings revealed no additional carcinomas.  26.5% of the women were at high risk because of a family or personal history of breast cancer. | Supplemental breast US increases the cancer detection rate in women with dense breast tissue (ACR categories 2-4). | 3b |
|  | Period studied: | 1/1995 – 9/2000 |  |  |  |  |
|  | US-Performer: | n=1 , specialized physician |  |  |  |  |
|  | US frequency: | 5-10 or 5-12 MHz |  |  |  |  |
|  | Scoring | 1. normal, 2. benign, 3. suspicious (biopsy) |  |  |  |  |
|  | US criteria: | Not given |  |  |  |  |
|  | Follow-up: | For benign 12 months |  |  |  |  |
| Leconte et al.,  2003,  Belgium | Data collection: | Prospective | n= 4236 of n = 5376 within the period studied underwent mammography and ultrasound in the same medical center. | Results can not be differentiated and assigned to separate groups of patients (73% screening, 24% follow-up, 3% palpable findings).  Results show that cancer yield for mammography is significantly lower in ACR 3 and ACR 4 breast tissue than in ACR 1 and 2. Objectivity of breast density classification (Inter-observer agreement): K= 0.91 | Breast Ultrasound is particularly useful for detection in dense breast tissue (ACR categories 3- 4). | 3b |
|  | Period studied: | 4/2000-3/2001 |  |  |  |  |
|  | US-Performer: | n=2, specialized physician |  |  |  |  |
|  | US frequency: | 7,5MHz |  |  |  |  |
|  | Scoring | 1. negative, 2. typical cyst, 3. all other |  |  |  |  |
|  | US criteria: | Specified |  |  |  |  |
|  | Follow-up: | No data |  |  |  |  |

### *LoE = Level of Evidence according to Oxford classification diagnostic studies
