# Supplementary material for: Early detection of breast cancer: benefits and risks of supplemental breast ultrasound in asymptomatic women with mammographically dense breast tissue. A systematic review
Source: BMC Cancer. 2009 Sep 20;9:335. doi: 10.1186/1471-2407-9-335 (PMC2760575; doi:10.1186/1471-2407-9-335)
Supplement: Additional file 3 — Evidence table of individual studies on supplemental ultrasound in breast cancer screening (Part II). Detailed analysis of parameters for diagnostic accuracy. [file 1471-2407-9-335-S3.DOC]

| **Table 3: Evidence table with results of individual studies on supplemental ultrasound in breast cancer screening** (Part II) | | | | | |  | |
| --- | --- | --- | --- | --- | --- | --- | --- |
| **Author/ year/**  **country** | **Age** | **Population, ultrasound scoring details, malignant histology and tumor size** | | **Positive predictive value (PPV) for malignancy scored by ultrsound** | **Biopsy rate because of ultrasound, positive predictive value (PPV) of biopsy** | **Cancer yield for ultrasound of all cancer detected, cancer yield for ultrasound within study population** | **ACR- Breast density examined / cancer detection rate per category** |
| Buchberger  et al.,  2000,  Austria | Median age. 47.6 years | Scoring n= 405  1. benign n= 113  2. indeterminate n= 167  3. malignant n= 125 | Malignant histology  1. n= 0 2. n= 2 3. n= 35   (mean size 9.1mm) 35 invasive, 2 non-invasive  N0 not stated) | PPV: 28% | 3.3% **(**269/8103)  PPV for biopsy: 13.75% (37/269) | 23%,  0.41% | ACR 2-4  ACR 2: 0.4% (2/979),  ACR 3: 0.3% 24/6916),  ACR 4: 1.1% 12/1075) |
| Corsetti et al.,  2008,  Italy | Mean age 52 years | Scoring n =9144 of 9157  US BIRADS no details | Malignant histology no details  (median size not given in mm:  n =1/pTis,  n = 3/pT1a,  n = 20/pT1b,  n = 10/pT1c,  n = 3/ >pT2)  36invasive, 1 non-invasive  N0=86.5% | PPV: not determined | 4.7% (436/9144)  PPV for biopsy: 8.4% (37/436) | 18.2% (37/203),  0.40% (37/9144) | ACR 3+4  Cancer detection not stated for separate categories |
| Crystal et al.,  2003,  Israel | Median age 52.1 years, SD 8.1 years. | Scoring n =1199 average risk  (n = 1517 including high risk):  1. normal n=1115 (n=1427)  2. benign n=69 (n=71)  3. indeterminate n= 12 (n =14)  4. malignant n= 3 (n=5) | Malignant histology:  1. and 2: n =0  3. (n = 2)  4. n= 3 (n = 5 )  mean size: 11mm  (median size 9.6mm), all invasive  N0=86% | PPV: 20 % | 2.3% (28/1199)  PPV for biopsy: 10.7%(3/28) | % could not be determined, 0.25% (3/1199) | ACR 2-4  ACR 2: 0%,  ACR 3: 0.4%,  ACR 4: 0.1% |
| Kaplan, 2001,  USA | Age range: 35-87 years | Scoring n =1862:  1. negative: n = 1612  2. positive: n = 250 | Malignant histology:  1. 0%  2. 2% (5/250)  (median size 9 mm, 6-14mm all invasive)  N0 = 100% | PPV: 2% | 2.7% (51/1862)  PPV for biopsy: 9.8% (5/51) | % could not be determined, 0.26% (5/1862) | ACR 2-4  Cancer detection not stated for separate categories |
| Kolb et al.,  2002,  USA | Median age 59.6 years SD 15.8 years | Scoring n = 12193 (n = 13547 all ACR 2-4)  1. negative n= 11432 (n =12748)  2. benign n= 441 (n = 441)  3. suspicious n = 320 (n= 358) | Malignant histology:  1. and 2. n =0  3. n = 37  (median size 9.9mm, 36 invasive, 1 noninvasive)  N0= 89% | PPV: 10.3% | 2.7%  PPV for biopsy: 10.3% | 15% relative, 0.23% overall | ACR2-4  ACR 2: 0.11%,  ACR 3: 0.27%,  ACR 4: 0.25% |
| Leconte et al.,  2003,  Belgium | Median age: 60.7 years, age range 41-87 years | Scoring n =4236 no details | Malignant histology :  n= 16  (median size 10.9 mm;13 invasive, 3 noninvasive)  N0 not stated | PPV: not determined | Biopsy rate not given,  PPV for biopsy: not determined | 34% (16/47) 0.37% (16/4236) | ACR1-4  ACR 1 + 2: n= 5, ACR 3 + 4: n= 11 |
